# How to Treat and Reconstruct the Pelvis After Chondrosarcoma Resection: Indications and Results at Medium- and Long-term in a Series of 34 patients

**DOI:** 10.1007/s13193-025-02408-3

**Published:** 2025-10-01

**Authors:** Daphne Sorrentino, Giovanni Zoccali, Francesca Sperati, Carlo Maria Bruno, Luca Paolucci, Dario Attala, Carmine Zoccali

**Affiliations:** 1 Department of Anatomical,Histological Forensic Medicine and Orthopedic Science, University of Rome, Piazzale Aldo Moro 5, 00185 Rome, Italy; 2 Plastic and Reconstructive Unit, IRCCS – Regina Elena National Cancer Institute, Via Elio Chianesi 53, 00144 Rome, Italy; 3 Statistical Unit, IRCCS – Regina Elena National Cancer Institute, Via Elio Chianesi 53, 00144 Rome, Italy; 4 Oncological Orthopedics Department, IRCCS – Regina Elena National Cancer Institute, Via Elio Chianesi 53, 00144 Rome, Italy

**Keywords:** Pelvic chondrosarcoma, Pelvis tumor, Pelvis resection, Pelvis reconstruction, Allograft, Custom-made prosthesis

## Abstract

Pelvic chondrosarcomas (CS) present complex diagnostic and therapeutic challenges due to their insidious onset and deep anatomical location. This study analyzes clinical, surgical, oncological, and functional outcomes in a consecutive series of 34 patients treated with wide resection, with the aim of proposing a surgical decision-making flowchart based on tumor localization. All patients with pelvic CS treated with wide resection between February 2005 and December 2021 were retrospectively reviewed. Epidemiological data, imaging, surgical details, histology, complications, oncologic outcomes, and functional results were collected. Tumor localization followed the Enneking and Dunham classification. Preoperative biopsy underestimated tumor grade in over 50% of cases. Wound infection was the most common complication (64.7%) and leading cause of perioperative mortality. Surgical Duration correlated with complication risk. Five-year overall survival was 60.2%, significantly influenced by age and histological grade. Local recurrence occurred in 10.3% of cases, associated with higher-grade tumors. Functional outcomes varied by resection site, with better scores in type I and III resections. Reconstruction strategies were adapted to anatomical site, tumor extension, and patient condition. Histological grade and age are key prognostic factors in pelvic CS. Wide resection remains essential due to frequent underestimation of tumor aggressiveness. Surgical approach and reconstruction should be tailored to tumor location to optimize oncologic control and functional outcome. A flowchart is proposed to guide surgical planning. Larger, more specific series are recommended to attain statistical significance and validate our findings and protocol.

## Introduction

Chondrosarcoma (CS) is a rare primary bone tumor characterized by its cartilaginous differentiation. In its primary form, it originates from normal tissue, whereas in secondary forms, it arises from preexisting benign bone tumors such as enchondromas or exostoses [1].

The primary treatment modality is surgical, except for cases of dedifferentiation where chemotherapy becomes indicated, often in conjunction with surgery, or in instances of systemic dissemination [1].

Although CS can develop in various skeletal sites, pelvic CS poses unique diagnostic and therapeutic challenges due to the complex anatomy and proximity to critical neurovascular and visceral structures [2].

It is relatively common, accounting for approximately 22–39% of all cases; often, pelvic CS presents insidiously, with nonspecific symptoms such as localized pain, swelling, or discomfort in the pelvic region. As the tumor grows, patients may experience pain radiating to adjacent areas, pelvic pressure, and even neurological deficits. The vague and subtle nature of these symptoms often leads to delayed diagnosis, contributing to the advanced stage of disease at presentation [1, 2].

En-bloc wide resection is the cornerstone of treatment for pelvic CS, aiming to achieve complete tumor excision while preserving pelvic function. The extent of resection depends on tumor size, location and involvement of adjacent structures. In cases of undifferentiated tumors, chemotherapy maybe considered to facilitate surgical resection and improve local/systemic control [1, 2].

Surgical procedures exhibit significant variability contingent upon specific tumor localization: CSs confined to the iliac wing, allowing for resection without disrupting the pelvic ring, afford a relatively straightforward surgical approach, correlating with a more favorable prognosis. Conversely, tumors occupying the sacroiliac joint and acetabular regions necessitate more intricate excisions, entailing a heightened surgical demand and posing a risk of intralesional transgression, thereby contributing to a less favorable prognosis. Moreover, the reconstruction process becomes more intricate in such cases, often resulting in diminished residual function.

Given the paucity of specific information in the literature, the primary objective of the present paper is to analyze the outcomes of a consecutive series of 34 patients afflicted with pelvic CS. This analysis entails the reporting of indications, complications, and survival rates associated with both tumor grade and precise localization. The secondary aim of this study is to establish a comprehensive flowchart designed to provide guidance for the surgical treatment approach.

## Materials and Methods

All patients with pelvic CS of any grade who underwent wide surgery, regardless of the type of reconstruction, from February 2005 to December 2021 were evaluated. Patients who underwent Inter-Ileo-Abdominal Amputation (IIAA) due to disease location or clinical situation were also included. The presence of distant metastases was considered an exclusion criterion, as well as patients with a follow-up of less than a year and those with unclear or incomplete data.

Data acquisition was conducted by consulting patients’ medical records, which encompassed radiological investigations. Pertaining to each patient, epidemiological and clinical dataset was collected from the presentation to the last follow-up.

Diagnoses were established via CT-guided biopsies in all instances, except for cases that came under observation subsequent to an initial misguided approach in a non-specialized facility. In such cases, diagnosis was based on the specimen obtained during the initial surgery. Histological evaluations were performed by a pathologist specializing in musculoskeletal oncology.

The Enneking staging system was used to grade the tumors and describe their tumor extension [3].

The patients underwent local evaluation using gadolinium-contrasted MRI to assess tumor extension and involvement of soft tissues. Pelvic X-rays and CT scans were employed to evaluate bone involvement. Furthermore, 18-FDG PET/CT was employed to assess local metabolism and identify potential distant metastases, while a total-body CT scan substituted for this in certain cases.

The Enneking and Dunham classification was employed to characterize tumor localization [4]. Each case was deliberated upon in a multidisciplinary meeting to determine the optimal approach.

All patients underwent pelvic resection surgery and reconstruction or IIAA, based on preliminary histological and imaging studies, as well as the patient's overall condition considering age, comorbidities, and the overall potential for recovery following the surgical procedure.

For procedures conducted in multiple surgical stages, the last surgery's total duration was considered.

Surgical margins were classified according to Enneking’s definition, which defines a margin as “wide” when the tumor is resected with a cuff of healthy tissue and no microscopic tumor is present at the resection border. All surgical specimens were evaluated by a dedicated musculoskeletal pathologist, who confirmed the absence of tumor at the inked margins.

To ensure adequate margins, preoperative planning included high-resolution MRI and 18F-FDG PET/CT imaging, and all cases were reviewed by a multidisciplinary tumor board. Intraoperatively, en bloc resection often required the deliberate sacrifice of adjacent neurovascular or osseous structures, such as sacral roots or pelvic vessels, when direct involvement was suspected or confirmed.

In cases where the tumor was closely adherent to major vascular structures, a wide margin was still pursued through meticulous intra-adventitial dissection. In these situations, the adventitial layer of the vessel was intentionally left attached to the tumor, enabling en bloc resection with a minimal, but oncologically safe, soft tissue cuff.

Conversely, in the rare cases where a wide margin was judged technically unachievable due to extensive neurovascular involvement or endopelvic infiltration, a primary IIAA was planned and performed upfront, in order to ensure oncologic adequacy and avoid the risks associated with an inadequate resection.

Complications were classified as early or late based on whether they manifested before or after 60 days following the index surgeries, respectively.

Wound infection was defined based on the presence of dehiscence, regardless of the local isolation of bacteria.

Anemia was defined as blood loss necessitating only transfusion support, while hemorrhage referred to blood loss requiring revision surgery and surgical hemostasis to halt bleeding.

Oncological results are reported by evaluating Disease-Free Survival (DFS), Local Recurrence-Free Survival (LRFS), Distant Metastasis-Free Survival (DMFS), the Overall Survival (OS) and the Disease-Related Survival (DRS).

The Musculoskeletal Tumor Society Score (MSTS) and the Toronto Extremity Salvage Score (TESS) for the inferior limb were employed to evaluate patient residual function and appraise the functional outcomes [5, 6]. Patients who underwent IIAA were not included in the functional outcome analysis.

### Statistical Analysis

Descriptive statistics were employed to summarize the epidemiological characteristics of the study population. Categorical variables were presented using their absolute and relative frequencies, while continuous variables were represented by means and standard deviations (SD). The normality of continuous variables was assessed using the Kolmogorov–Smirnov test. To assess differences among continuous variables, the Mann–Whitney or Student’s *t*-test was utilized based on the nature of data distribution. Relationships between categorical variables were examined using Pearson’s Chi-square test. Survival curves were estimated and compared using the Kaplan–Meier product-limit method and the log-rank test. A *p*-value of < 0.05 was considered statistically significant. All statistical analyses were conducted using SPSS version 21.0 (SPSS Inc., Chicago, Illinois, USA).

The clinical studies adhered to the principles outlined in the Declaration of Helsinki; all patients provided consent for the publication of their data in an anonymized format prior to their inclusion.

Approval for the conduct of the current study was granted by the Local Ethics Committee.

All patients gave their informed consent to be included in the present study and for publishing of the present paper.

## Results

A series of 34 patients were analyzed, including 16 females and 18 males, with a mean age of 52.1 years (median: 51 range: 21–75). Epidemiological and clinical characteristics are summarized in Table 1.

**Table 1:** Table Epidemiological and clinical characteristics

Parameters	Value
**Patients**	34 (18 M – 26F)
**Age**	Mean: 52,1 years (range 21–75, median 51)
**Enneking Classification**	
Intracompartmental	
*IA*	*13*
*IIA*	*3*
extracompartmental	
*IB*	*9*
*IIB*	*7*
*III*	*0*
**Symptoms**	
*Mass and swelling*	*15 whereof 13 with pain*
*Pain without mass*	*13*
*Neurological deficit*	*3*
*No symptoms*	*3 (accidentally discovered)*
**Diagnosis**	
*G1*	*8*
*G2*	*14*
*G3*	*8*
*Dedifferentiated*	*3*
*Mesenchymal*	*1*
**Preexisting conditions**	
*Exostosis*	*7 whereof 3 hereditary multiple exostoses*
*Previous curettage at a non-specialized center*	*1*
*Hip replacement in acetabular CS*	*1*
**Disease localization following the Enneking and Dooham classification**	
*Area 1*	*3*
*Area 2*	*10*
*Area 3*	*5*
*Area 4*	*3*
*Area 1–4*	*8*
*Area 1–2-3*	*1*
*Area 2–3*	*4*
**Tumor Volume**	*Mean 3912,4 cc (range: 33,5–31,415,9)*
**Blood Units transfused during hospitalization**	*Mean 17,5 Units (range 0–83, median 11)*
**Hospital inpatient time**	*Mean 78,7 days (range: 8–306, median 46,5)*
**Follow-up**	
***All patients***	*Mean 46.7 months (range 1–134, median: 27.5)*
***Excluding patients who died from postoperative complications and those lost to follow-up (27 patients)***	*Mean 58.0 months (range 12–134, median: 52)*
***Surviving patients***	*Mean: 52.8 months (range 12–124, median: 49.5)*

Surgical Procedures (Following Enneking and Dunham’s classification):

In the three cases where CS was localized in area 1, resection of the iliac wing was performed without interrupting the pelvic ring, obviating the need for reconstruction.

In 14 patients (10 in area 2 and 4 in area 2–3), an acetabular resection was performed, extra-articular in four cases. Reconstruction was carried out as follows: custom-made hemipelvis prosthesis combined with hip arthroplasty in six cases, hip arthroplasty with an iliac stem cup in three cases, homoplastic graft with screws and hip prosthesis in one case, xenoplastic bone with plate and screws in two cases, autologous bone harvested from the residual iliac wing with screws in one case. In the last case, reconstruction was not performed due to the patient's clinical and social conditions.

In the five cases in area 3, resection of the inner portion of the obturator foramen was performed in two cases, resection of the ileo-pubic branch in one case, IIAA in one case, and resection of the ileo and ischio-pubic branches in another case. No orthopedic reconstruction was carried out. However, in the last reported case, a Gore-Tex membrane was used to restore containment to the abdominal viscera.

In the eight cases of CS in areas I–V, sacroiliac resection was performed. In one case, three lumbar hemivertebrae were also excised en-bloc [7]. Reconstruction was carried out in five cases using bone grafts (four homoplastic and one autologous), a rod, and screws placed between the spine and the remaining portion of the ischium. In one case, a custom-made prosthesis connected to vertebral stabilization was used. Reconstruction was not performed in the last two cases.

In two out of the three cases located in area 4, the pelvic ring was not disrupted, thus no reconstruction was necessary. The last case underwent an IIAA.

In cases where CS involved areas 1–2-3, acetabular and iliac wing resection was performed. Reconstruction was carried out using a homologous massive graft, plate, screws, and hip prosthesis.

### Surgery Duration

Average 503 min (median 513, range: 60–917); in 28 patients, a single surgical procedure and a single approach were necessary to remove the lesion. In five cases (four sacroiliac resections and one case of acetabular resection with ischium), two surgical stages were required, performed on two different days: the first for preparation and the second for tumor removal. In the last, most complex case, four surgical stages were necessary: an extra-anatomical suprapubic bypass was performed as the iliac vessels were inseparable from the disease, followed by spinal stabilization, anterior isolation of the disease, and finally lateral tumor removal [7].

### Surgical Margin

The margin was wide in all cases.

### Postoperative Diagnosis

The preoperative grade was confirmed in 15 cases (44.1%); in 17 cases, the postoperative diagnosis revealed a more aggressive pathology, with an increase of one grade in 14 cases, two grades in two cases, and three grades in the remaining case; in one case, a patient initially diagnosed with dedifferentiated CS based on biopsy underwent a new diagnosis of grade 3 CS; the last case diagnosed as a not well identified high-grade sarcoma was better identified as mesenchymal CS.

### Early Complications

23 patients experienced early complications (67.6%); the most common complication was wound infection, occurring in 22 cases, of which one resolved with conservative therapy. The remaining cases underwent surgical debridement, resulting in healing in 15 cases (with hardware removal necessary in one case), chronic infection in one case, healing after IIAA in two cases, and three cases resulting in death (one case underwent IIAA due to sepsis, one due to hemorrhage from iliac vein rupture, and another due to sepsis) due to ensuing systemic complications. Vacuum-assisted closure (VAC) therapy was applied in seven cases, of which six achieved healing.

Considering the age of 52 as a threshold value, wound infection occurred in 52.9% and 82.3% of patients aged younger and older, respectively. However, this difference, although suggestive, did not reach statistical significance (*p* = 0.151).

Histological grade and tumor volume also did not show a correlation with the onset of early complications (*p* = 0.258 and *p* = 0.714, respectively).

There might actually be a certain correlation dependent on the Duration of the surgery, as the average value in patients without early complications was 401.4 ± 160.1, whereas the Duration in patients who developed complications was 552.3 ± 242.2 (*p* = 0.070).

Progressive anemia requiring surgical revision occurred in two cases, one of which was fatal due to acute rupture of the iliac vein likely caused by impingement on the custom-made prosthesis. Another case involved a patient who initially underwent wound revision, then IIAA due to infection, and subsequently succumbed to septic shock.

Furthermore, one case of dislocation necessitated surgical revision followed by IIAA due to infection.

*Late Complications* Evaluated in 30 Patients (Four of Whom Died Early): two cases of infection, one being erysipelas occurring a year after the surgical procedure and unrelated to it, which led to the patient's death due to sepsis; one case of chronic infection resulting from an acute infection that led to a fatal outcome; one case of abdominal hernia resolved through plastic surgery; one case of myocardial infarction, likely unrelated to the surgical procedure.

#### Oncological Results

These were evaluated in 29 patients, excluding the five patients who died in the immediate postoperative period.

**Fig. 1:**
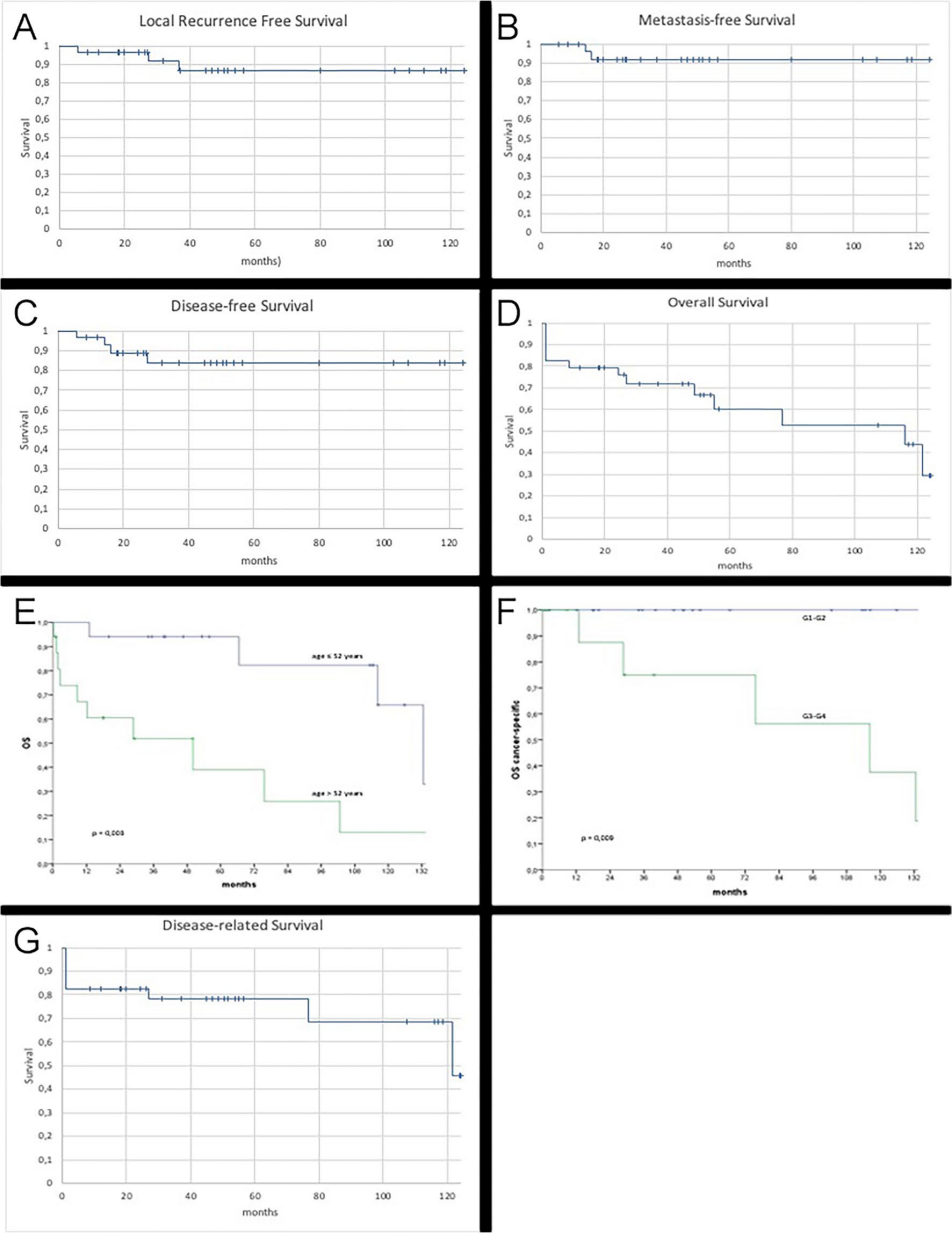
**A) **LRFS; the 5-year rate is 86,1%; **B) **DMFS curve; the 5-year DMFS is 92.0%; **C) **DFS curve; the 5-year DFS rate was 83.6%; **D) **OS curve; the 5-year OS rate is 60.2%; **E)** age-specific OS curves; **F)** grade-specific OS Curves **G) **DRS curve; the DRS rate is 78.2%

*Local Recurrence* occurred in three cases (10.3%), with a 5-year disease-free survival rate of 86.1%; the LRFS curve is shown in Fig. 1A.

Considering histological grade as a risk factor, local recurrence occurred in 3 out of 8 cases of grade 3/dedifferentiated CS (37.5%), and in none of the 19 cases with grade 1 or 2 CS; this difference was statistically significant (*p* = 0,0046). Age and tumor size also do not appear to influence the risk of local recurrence (*p*:0.999).

*Distant Metastases* occurred in two cases at 14.1 and 16.2 months after the surgery; both patients subsequently succumbed due to systemic disease progression. The first was afflicted with dedifferentiated CS, while the second had G3 CS. No metastases developed in patients with G1-G2 CS; however, the difference, although indicative, is not statistically significant (*p* = 0.086).

The 5-year metastasis-free survival rate was 92.0%; the Metastasis-Free Survival Curve is shown in Fig. 1B.

The 5-year disease-free survival rate was 83.6%; the disease-free survival curve is shown in Fig. 1C.

*Survival*: At an average follow-up of 46.7 months (range 1–134, median: 27.5), 14 patients have passed away, five of whom in the immediate postoperative period and nine subsequently. Of the five cases that died in the postoperative phase, two died from septic complications, one from acute bleeding and likely rupture of the iliac vein, one from progressive decline in performance status and multiorgan failure, unrelated to direct surgical or oncologic complications, and one from a cardiac event. Among the remaining cases, three died due to systemic disease progression (one with local recurrence as well), one due to progressive general health decline, and five due to issues unrelated to the disease.

The 5-year OS Rate was 60.2%; the OS Curve is shown in Fig. 1D.

OS was statistically correlated with both age (*p* = 0.003) and grade (*p* = 0.009); The Age-Specific OS Curves and the Grade-Specific OS Curves are reported in Fig. 1E and F, respectively.

Out of the 34 patients, two were lost to follow-up Due to changes in residence, 14 have passed away, and 18 are currently alive and seemingly disease-free, although there is suspicion of lung metastases in one of them.

The disease-related survival rate was 78.2%; the disease-related survival curve is shown in Fig. 1G).

*Functional Results* were assessed in 16 patients who were alive at the time of follow-up and had not undergone IIAA (two patients were excluded). The mean follow-up was 52.8 months (range: 12–124; median: 49.5 months).

The mean MSTS score was 70.0 (median: 70.0; range: 36.7–100), and the mean TESS score was 67.5 (median: 68.8; range: 22.3–99.1).

Functional outcomes varied according to the anatomical location of resection, classified by Enneking and Dunham:

- Area 1 (n = 3): MSTS 75.6%, TESS 76.1%;

- Area 1–4 (n = 2): MSTS 62.3%, TESS 55.8%;

- Areas 2 and 3 (n = 5): MSTS 60.0%, TESS 56.0%;

- Area 3 (n = 4): MSTS 91.6%, TESS 85.2%;

- Area 4 (n = 1): MSTS 40.0%, TESS 63.5%;

However, the differences observed between these groups did not reach statistical significance.

Better functional outcomes were observed in patients with G1–G2 chondrosarcomas compared to those with G3-dedifferentiated tumors, with mean MSTS and TESS scores of 71.8 and 70.5 versus 47.6 and 42.7, respectively. However, these differences, while indicative of a trend, did not reach statistical significance (*p* = 0.081 and *p* = 0.073).

Additionally, hip flexion limitation was noted in four patients, and limb length discrepancy was present in two cases, both of which were corrected with shoe lifts.

### Results for Specific Classes of Reconstructions

Outcomes for specific reconstruction techniques were analyzed only for custom-made prostheses in Enneking area 2 and for reconstructions performed with homograft, rod, and screws following sacroiliac joint resections in areas 4 and 4–1. The heterogeneity of the remaining reconstruction techniques did not allow for meaningful grouping or comparative analysis.

Among the six patients treated with a custom-made prosthesis, four developed wound infections: two resolved after surgical debridement, while two patients died, one due to sepsis and progressive deterioration of general health, and one due to acute rupture of the iliac vein.

In the remaining four patients, the mean MSTS score was 47.2%, and the mean TESS score was 51.5%. Among the four patients who underwent reconstruction with homograft, rod, and screws after sacroiliac joint resection, three experienced wound infections: two were successfully managed with debridement and revision surgery, and one evolved into a chronic fistula. One patient died from disease progression, and no functional data were available in that case. In the remaining three patients, the mean MSTS score was 62.2%, and the mean TESS score was 55.8%.

### Status at the Last Follow-up

Out of the 34 patients, two were lost to follow-up due to changes in residence. Fourteen have passed away, and eighteen are currently alive and seemingly disease-free, although there is suspicion of lung metastases in one of them.

*Difference Between Groups*: Patients were divided based on disease localization into four main groups with the same surgical technical characteristics (Table 2):

- Group 1: Patients with disease localized exclusively in area 1;
- Group 2: Patients with disease localized in area 2 (area 2, area 2–3, area 1–2-3);
- Group 3: Patients with disease localized exclusively in area 3;
- Group 4: Patients with disease localized in area 4 (area 4, area 1–4).

**Table 2:** The main characteristics observed in the specific groups

**Type**	**N°**	**Età**	**MG**	**BP**	**TV**	**SD**	**EC**	**WS**	**HD**	**UT**	**R**	**A**	**PD**	**Mets**	**MSTS/** **TESS**
1	3	45,3	1,6	66,7%	3638	203	0	0	12	2,3	0	0	0	0	75,6/76,1
2, 2–3, 1–2-3	15	58,3	2,4	40%	1266	644	80,0%	66,7%	90	13,7	2	2	4	1	56,2/54,7
3	5	47,0	1,8	40%	4107	408	25%	25%	21	6,6	0	1	1	0	91,7/85,2
4, 1–4	11	47,6	2,3	35,7%	7234	437	90,9%	90,9%	108	31,7	1	1	0	1	56,6/63,5

*MG Mean Grade; BP: Biopsy Predictivity; TV: Tumor Volume (cc); SD: Surgery Duration (minutes); EC: Early Complications; WS: Wound Suffering; HD: Hospitalization Duration; UT: Units Transfused during Hospitalization; R: Recurrence; A: Amputations; PD: Postoperative Deaths; Mets: Distant Metastases*

## Discussion

The optimal treatment for low-grade CS remains debated [8]. While wide resection is universally recommended for high-grade lesions, some authors propose intralesional treatment for low-grade CS to preserve function, accepting a modest risk of recurrence [8]. However, preoperative grading is often unreliable, especially in pelvic tumors, due to intralesional heterogeneity and deep location, leading to frequent underestimation of tumor aggressiveness [9]. In our series, biopsy predicted the definitive grade in fewer than 50% of cases, with several instances of substantial upstaging, including one case reclassified as dedifferentiated CS. These findings support the rationale for wide resection even in low-grade CS of the pelvis, where local recurrence may be difficult or impossible to manage due to contamination of endopelvic tissues [10].

Several studies have highlighted the value of ^18F-FDG PET/CT in differentiating CS grades and identifying metabolically active regions. However, this modality remains insufficient to reliably distinguish low-grade CSs from benign enchondromas [11, 12]. Similarly, contrast-enhanced MRI shows limited ability to differentiate among less aggressive chondroid lesions [13, 14]. These limitations emphasize the need for improved imaging techniques to better guide biopsy targeting and enhance preoperative tumor grading.

From a technical standpoint, the treatment of pelvic CSs remains complex. Although low-grade lesions are more frequent and generally associated with favorable oncologic outcomes, functional results, particularly in tumors involving Enneking area II, are often unsatisfactory. This is largely due to the considerable tumor size at presentation and the extensive surgical procedures required for wide resection, often involving the sacrifice of critical load-bearing and joint structures.

The absence of effective systemic or radiotherapeutic options further emphasizes the role of surgical management in determining outcomes. In our series, nearly half of the patients presented with large tumors that were already symptomatic, though not yet causing compressive complications. Pain was the most frequent presenting symptom, while the deep location within the pelvis often contributed to delayed diagnosis.

The anatomical site and extent of muscle and nerve involvement were key determinants of postoperative function. However, due to the limited sample size and heterogeneity of reconstruction strategies, definitive conclusions regarding the functional superiority of specific techniques cannot be drawn.

**Fig. 2:**
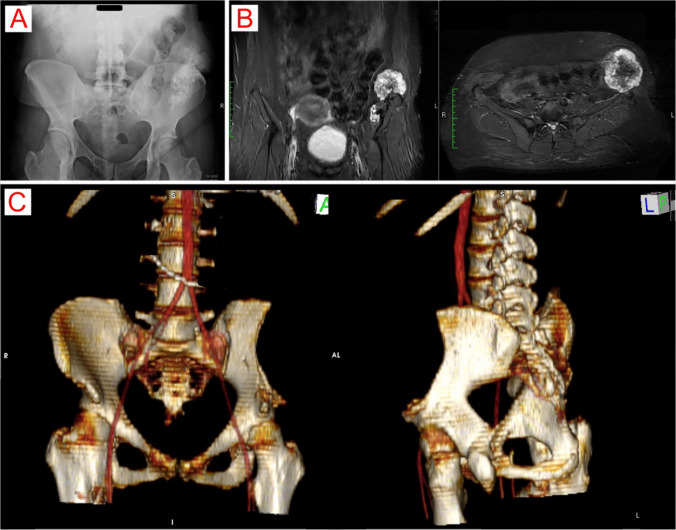
Thirty-eight-year-old female patient with Grade 2 CS of the left iliac wing arising from exostosis in the context of multiple hereditary exostosis; Preoperative X-ray **(A)** and STIR-MRI **(B)** showing the lesion; **C**) 3D reconstruction after removal of the lesion

Tumors located in area 1 are typically peripheral, arising from exostoses and of low grade (Fig. 2A–B); from a technical standpoint, the excision procedure is easily performed, and the Duration is relatively short; in our case series, an average Duration of about 200min was observed; within the limitations of the small sample size, this duration is nevertheless justified by the distance these lesions maintain from the vascular-nerve bundles and the lack of reconstructive time, sometimes limited solely to abdominal wall reinforcement, necessary to reduce the risk of hernia formation. Consequently, postoperative complications are also low (Table 2).

The functional consequences are relative and arise from the muscular sacrifice of the gluteal muscles on the outer side and the iliac muscles on the endopelvic side, directly linked to the size of the neoplasm; the intact pelvic ring justifies the absence of reconstruction (Fig. 2C).

Indeed, among the three patients who underwent type 1 resection, two are able to walk without the use of crutches, while the third requires one crutch. It's important to note that the latter is also affected by multiple exostoses syndrome, which directly impacts her functional capacity.

**Fig. 3:**
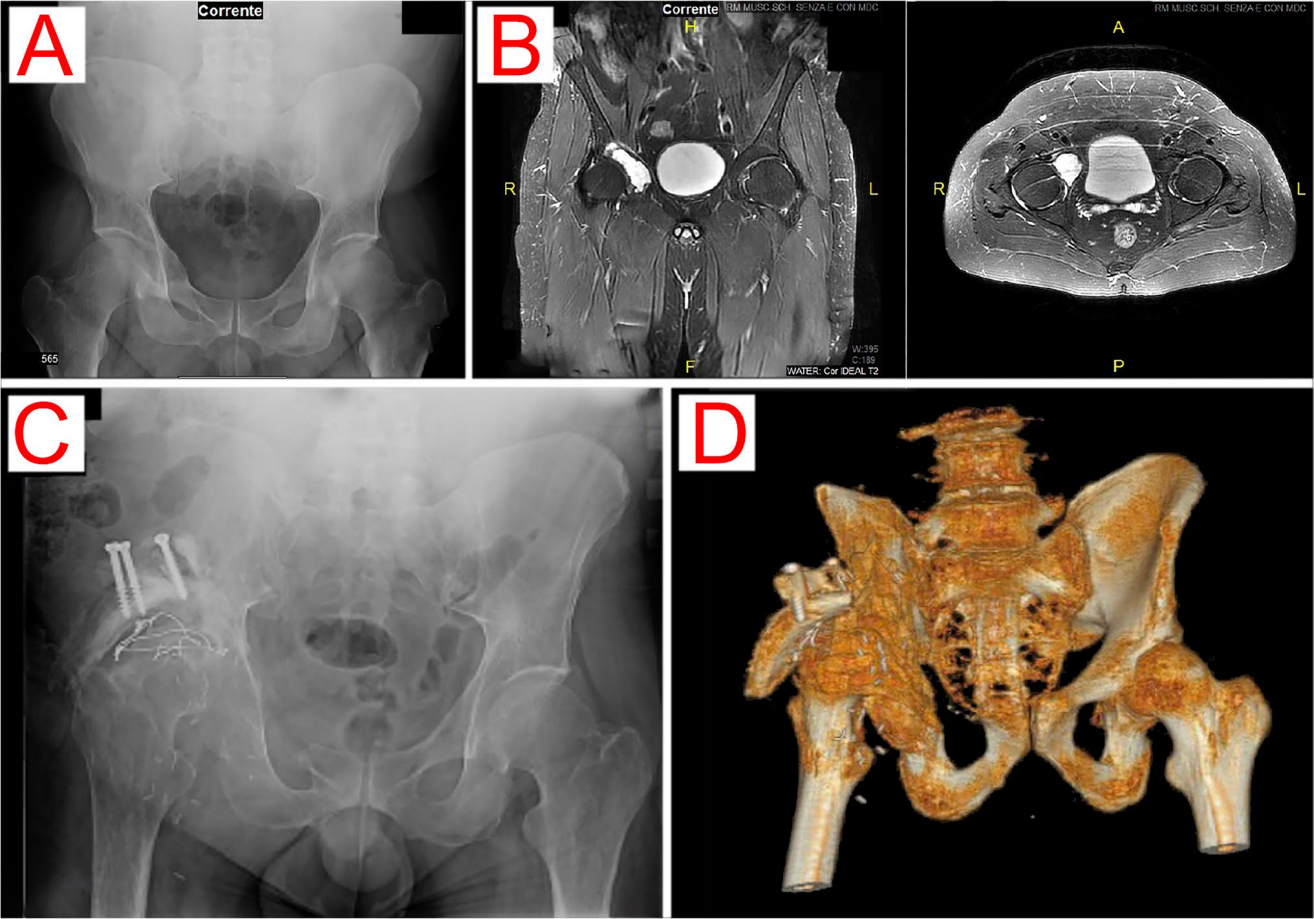
Fifty-three-year-old male patient with Grade 2 CS of the acetabulum (Enneking and Dunham’s area 2); preoperative X-ray **(A) **and STIR-MRI **(B)** highlights an acetabular osteolytic area with widening of the bone profile; **C** and **D)** postoperative X-ray and CT scans

Tumors located in area 2 are among the most technically demanding due to frequent joint involvement, which leads to biomechanical instability and necessitates reconstruction to restore both pelvic ring integrity and hip joint function (Fig. 3). Reconstruction is most commonly performed with custom-made titanium prostheses, although massive allografts combined with prosthetic components are also viable alternatives [15].

In our series, the average surgical Duration for area II resections was 632min, reflecting the complexity of the procedure. This was associated with a high rate of early complications (78.6%), primarily wound infections (71.4%), which were the leading cause of early postoperative mortality (Table 2). The extensive Enneking approach often requires large gluteal flaps, which may be hypovascularized and prone to dehiscence and infection. Mortality within the first month occurred in four patients (28.6%): three due to sepsis and one due to hemorrhage. Notably, deceased patients had a higher mean age (70 years) compared to survivors (53 years), suggesting a potential age-related risk factor.

Due to the high complication rate observed, particularly in elderly or comorbid patients, we have progressively adopted a more selective approach to reconstruction, favoring prosthetic implantation only in patients with optimal local tissue conditions and general health.

Despite adequate reconstruction, functional outcomes remain modest Due to frequent muscle sacrifice, particularly of the iliopsoas and surrounding pelvic stabilizers, resulting in impaired hip flexion. When resections extended into area III, the mean MSTS and TESS scores were 56.2% and 54.7%, respectively. Additionally, the ileo-inguinal approach has been associated with an increased risk of lower-limb lymphedema, likely due to lymphatic disruption in the inguinal region, although specific data on incidence remain limited.

Comparable literature supports these findings. Vahabi et al. reported a mean MSTS of 60.0% after composite reconstruction with irradiated autograft and total hip arthroplasty [16], while Bus et al. described a mean MSTS of 70.0% following reconstruction with cone prostheses—an approach that requires adequate residual iliac bone stock [17].

Tumor resection in Enneking area 3 interrupts the pelvic ring but does not compromise axial load transfer between the spine and lower limbs, thus generally obviating the need for structural reconstruction. Despite requiring careful isolation of the iliofemoral neurovascular bundle, the procedure is typically shorter than resections in area II, with an average Duration of 408min and a low postoperative complication rate (Table 2). Only one of five patients developed a wound infection, successfully managed with surgical revision.

Functional deficits mainly stem from potential compromise of the iliopsoas and detachment of the thigh adductors. Given the iliopsoas’ pivotal role in hip flexion—owing to its passage over the ilio-pubic ramus—its loss results in posteriorization of the tendon and a shift toward an adduction function. In high-grade CSs, the proximity to major pelvic vessels may necessitate an IIAA to ensure oncologic margins; one such case in our series resulted in death from myocardial infarction.

Among survivors, functional outcomes were satisfactory: all patients achieved independent ambulation, with only one reporting residual adduction difficulties and another requiring occasional crutch use for prolonged walking.

**Fig. 4:**
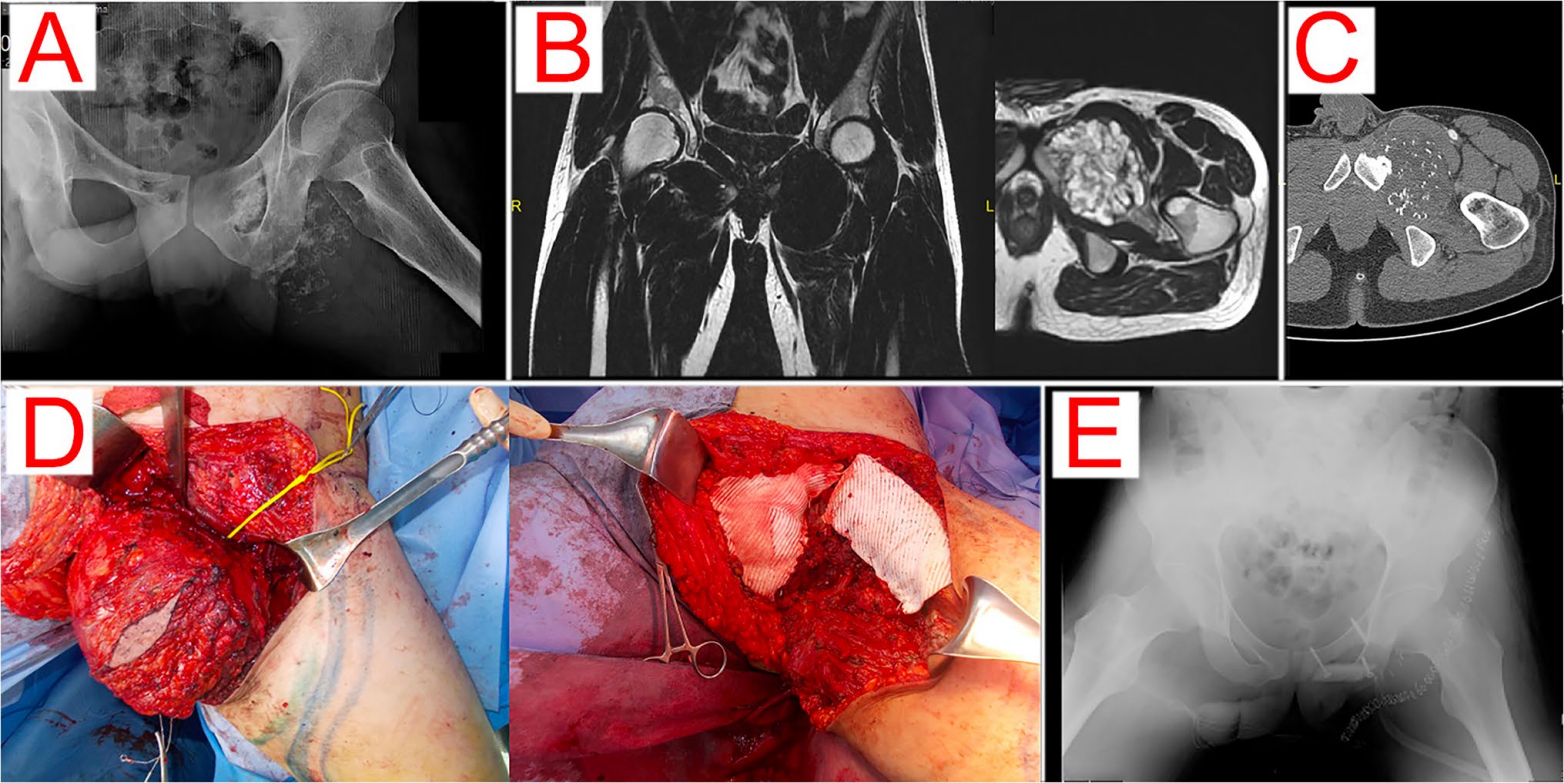
Twenty-six-year-old male patient with Grade 1 CS of the obturator foramen. **A)** Preoperative X-ray revealing calcifications within the matrix; **B)** coronal T1-weighted MRI on the left showing a large hypointense mass; axial T2-weighted MRI on the right displaying hyperintense signal; **C)** CT scan clearly depicting calcifications within the matrix; **D)** intraoperative photos of the large inguinalmass (biopsy scar in the foreground) on the left and reconstruction using homoplastic bone reinforcement and Gore-Tex membrane on the right; **E)** postoperative X-ray

Reconstruction in this area was typically limited to repairing the inguinal ligament and supporting abdominal viscera, using either homologous fascia lata or Gore-Tex mesh anchored between the pubic symphysis and acetabulum (Fig. 4).

**Fig. 5:**
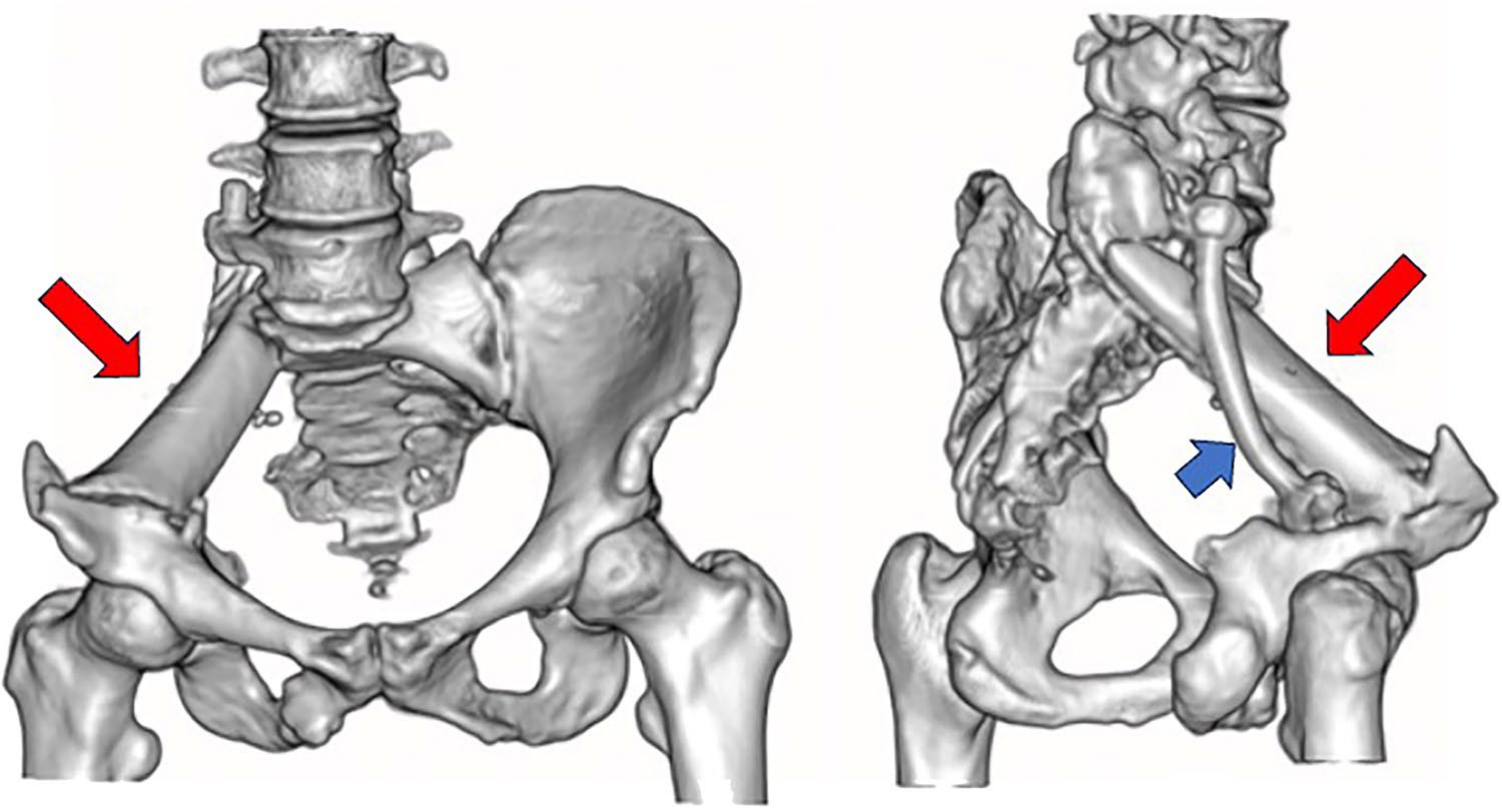
Technique for reconstruction of the pelvic ring after resection of the sacroiliac joint; an allograft tibia (red arrow) is positioned between the remaining iliac and sacral remnants; primary stability is ensured by a bar connecting two screws inserted in the ischium and the pedicle of L5 (blue arrow)

**Fig. 6:**
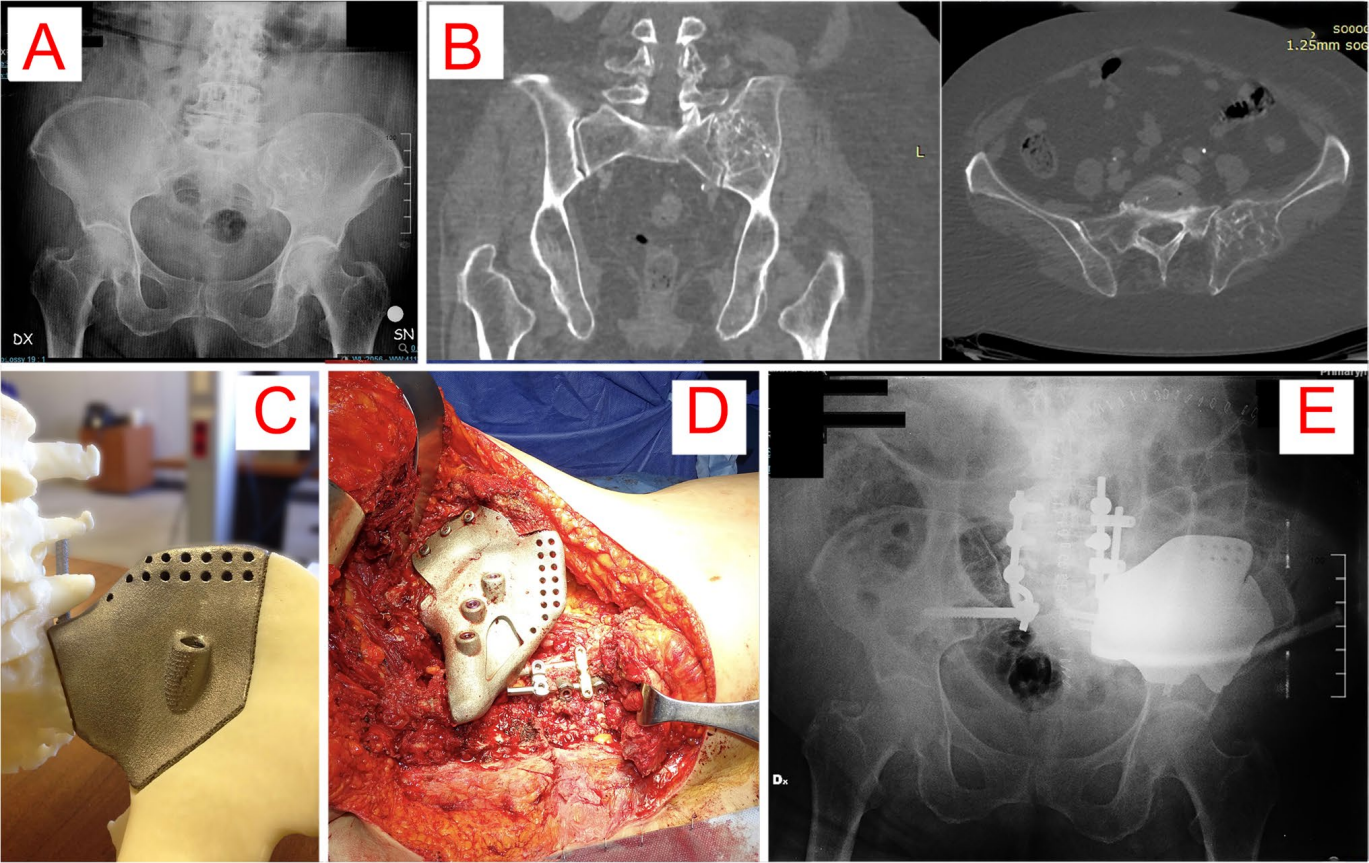
Seventy-three-year-old woman affected by grade 2 CS of the iliac side of the sacroiliac joint; in **(A)** the preoperative X-ray highlights the osseous rearrangement and calcifications more clearly seen in CT scan **(B)**. In **(C)** a preoperative model of the custom-made prosthesis designed for reconstruction; in **(D)** an intraoperative photo of the reconstruction with the sacroiliac prosthesis

Tumors located in area 4 are particularly demanding due to the anatomical proximity of major vascular structures and the sacral roots, which increases the technical complexity and potential risk of complications. Therefore, it is often necessary to perform an anterior preparation step to separate them from the disease by inserting a temporary protective spacer, followed by a posterior resection step to remove the tumor [18]. The wide excision of such tumors often involves the disruption of the pelvic ring, as well as the potential compromise of the L5 root that runs along the proximal surface of the sacral wing, and the sacral roots [19]. They can be of considerable size and compromise a more or less variable portion of area I, further exacerbating the resulting mechanical instability. The reconstruction of the pelvic ring, although some authors have expressed opposing views, advocating for improved functionality and fewer complications, becomes more significant as the interruption extends, particularly to reduce the rise of the hemipelvis and the development of secondary scoliotic deformities of the spine. Various techniques are described in the literature, including the use of vascularized fibular flaps, allografts, custom-made prostheses; our institution has recently proposed the use of an allograft from the tibia stabilized with transpeduncular screws and connecting bars, achieving a stable reconstruction of the pelvic ring and functionality related to the sacrifice of the structures involved in the tumor (Fig. 5) [20]. Reconstruction with custom-made prostheses is another valid alternative that theoretically should allow for reduced surgical times and a lower risk of infection. However, the presence of large metal surfaces still represents a risk factor. In the case of custom-made prostheses, a connection to a vertebral stabilization system may be necessary to neutralize shear forces at the bone-prosthesis interface (Fig. 6).

Interventions in areas 1–4 have an average Duration of 437min and are burdened with a high percentage of postoperative complications (Table 2); out of the 11 treated cases, complications occurred in all cases except one; wound infection occurred in 10 cases, and surgical revision was performed in all cases, resulting in healing in 8 of them and chronicization in one case; in the last case, an IIAA was required due to septic complications; the patient subsequently died due to disease progression.

### Complications

Pelvic resection surgery for CS is associated with a high rate of complications, particularly in resections involving area 2 and the combined area 1–4 group (Table 2). The most frequent and severe complication is wound infection, occurring in 64.7% of cases. These infections can progress to periprosthetic contamination and sepsis, which represent the main indications for IIAA and the leading cause of postoperative mortality.

Several predisposing factors may contribute to wound complications, including devascularization of flaps from extensive surgical exposure, dead space formation, and proximity to abdominal viscera that increases the risk of contamination. Although not statistically significant, older age appeared to correlate with higher infection rates, possibly due to reduced muscle trophism and impaired tissue perfusion. Similarly, longer operative times were observed in patients who developed early complications (552.3 min vs. 401.4 min).

Various strategies can be considered to reduce the incidence of complications, particularly infectious ones. For example, the use of plastic flaps for coverage has the dual advantage of reducing tension on wound margins and simultaneously reducing dead space postoperatively [21].

The selection of a specific flap for reconstruction is contingent upon various factors, including patient characteristics, the condition of the donor site, the surgical approach employed, and the localization of the wound. In the case of defects located in Enneking areas 1 and 2, several flap options can be considered, including the anterolateral thigh (ALT) pedicled flap, deep inferior epigastric perforator (DIEP) flap, rectus abdominis flap, anteromedial thigh (ORAM) flap, and the vascularized rectus abdominis (VRAM) flap.

For defects situated in Enneking area 3, potential flap choices include the gracilis flap, perforator adipofascial flap (PAP), and the ALT pedicled flap.

Du et al. demonstrated that sartorius flaps combined with mesh reconstruction in area 3 significantly reduced hernia and infection rates [22].

Defects in Enneking area 4 can be addressed using the inferior gluteal artery perforator (IGAP) pedicled flap, superior gluteal artery perforator (SGAP) pedicled flap, and the lumbar artery perforator (LAP) pedicled flap.

Other strategies might include staged reconstruction with antibiotic spacers to reduce early contamination risk, although this requires a second procedure in a fibrotic surgical field [23].

Given the possible contribution of gut microbiota to surgical site infections, prophylactic ileostomy could be advocated in complex cases; However, this approach remains anecdotal and lacks validation through clinical studies.

Finally, emerging approaches such as antimicrobial-coated prostheses (e.g., iodine or silver coatings) [24] and localized antibiotic delivery systems [25] may help reduce biofilm formation and hardware contamination.

From a functional perspective, the residual function is closely linked to the extent of resection and the specific structures involved, such as the gluteus maximus, iliopsoas muscle, and affected nerves and roots. The average MSTS and TESS scores are comparable to those reported in area 2, highlighting the impact of these surgical interventions. While no consensus exists regarding the optimal reconstructive technique for each pelvic defect type, it appears reasonable to infer from clinical experience that functional outcomes are influenced by the extent of bone loss and the integrity of surrounding musculature and neurovascular structures.

Moreover, this study confirms the value of grade as a prognostic factor, both for local recurrence and survival, although statistical significance was not achieved.

Indeed, local recurrence occurred in 5.3% of cases of Grade 1–2 CS and in 37.5% of cases of Grade 3-dedifferentiated CS. Furthermore, no cases of Grade 1–2 CS developed distant metastases.

OS was thus statistically correlated with both patient age and grade, underscoring the importance of the latter in determining the outcome for these patients.

### Treatment Protocol

**Fig. 7:**
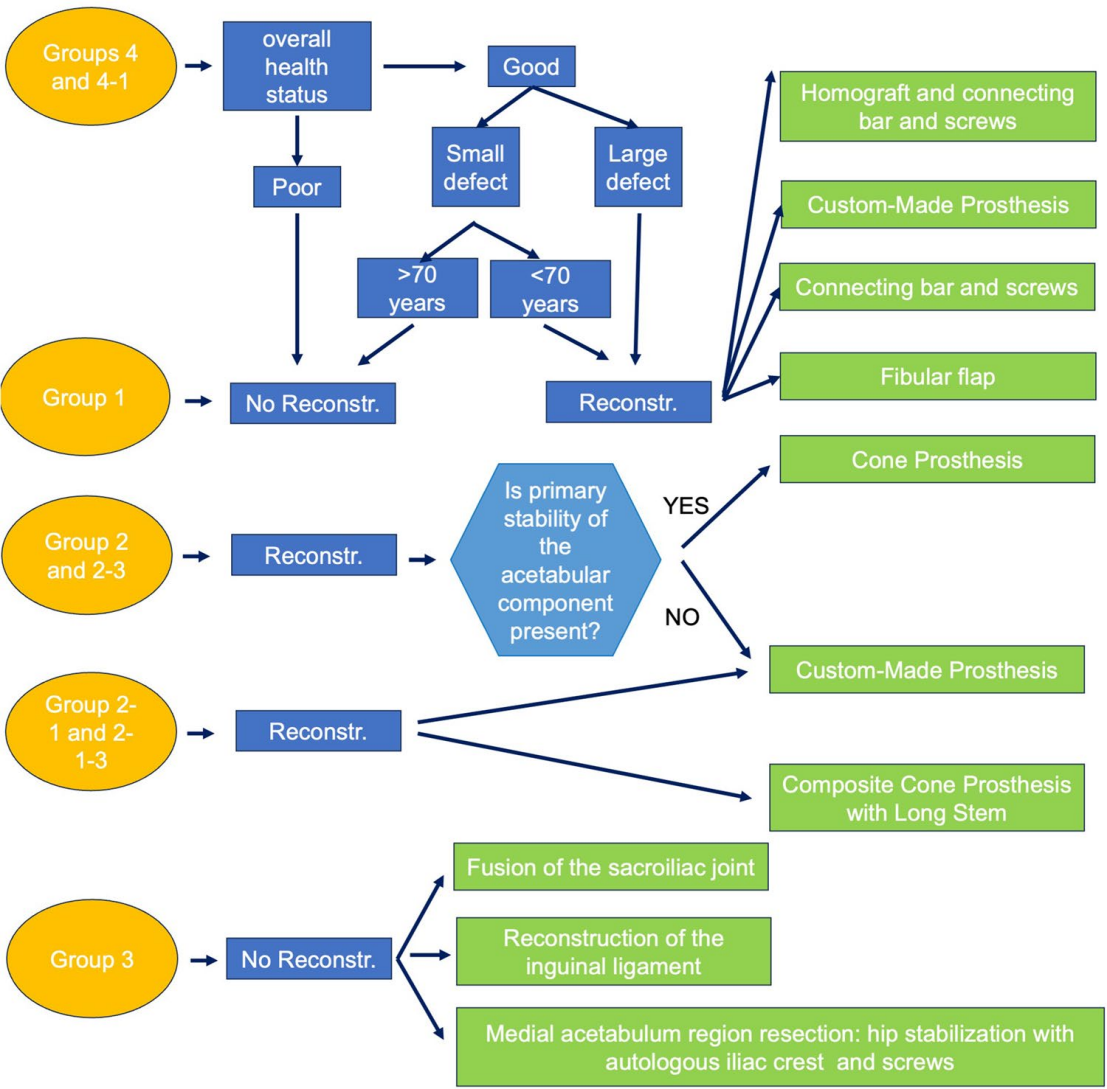
Flowchart summarizing the surgical reconstruction strategy following pelvic chondrosarcoma resection, based on Enneking classification, defect size, patient general condition, and intraoperative acetabular stability

 Based on the evaluated treatments, we propose the following treatment protocol, referring to the respective Enneking and Dunham areas, and categorizing patients into groups with similar reconstructive challenges after resection (Fig. 7).

Group 4 and 1–4: Tumors involving the sacral and iliac wings with pelvic ring interruption. Reconstruction is generally recommended due to mechanical instability, although some authors advocate against it to reduce complications [26].

Surgical indication should consider patient age, general health, and defect size. In elderly or frail patients with small defects, non-reconstruction may be acceptable, allowing the hemipelvis to rotate around the pubic symphysis. In younger, more active patients with larger gaps, reconstruction helps prevent symphyseal pain and spinal imbalance.

Reconstruction options include custom-made prostheses, bone homografts fixed with ischial and pedicular screws, or vascularized fibular flaps, which demonstrate good hypertrophic union but increase surgical time and complexity [20, 27].

Abdominal wall reconstruction is advisable to prevent herniation.

Group 1: Lateral iliac wing lesions that do not disrupt pelvic continuity. These typically require no skeletal reconstruction, only reinforcement of the abdominal wall when necessary.

Group 2: Periacetabular lesions requiring joint resection. Reconstruction is mandatory to restore joint mechanics, usually via hemipelvis prostheses anchored to the iliac bone [28].

If bone stock allows, cone prostheses are preferred for their primary stability. When the iliac base is insufficient, custom-made implants or massive allograft–prosthesis composites are required.

Extended Group 2–1: Involving extensive acetabular roof and iliac involvement. Custom hemipelvic prostheses are recommended. Composite reconstructions using structural allografts and cemented prostheses are an alternative. A long-stem cone prosthesis should always be available to ensure reliable fixation and bone integration.

Extending the resection of Group 2 to Enneking and Dunham’s area 3 does not alter reconstruction techniques, except for the possibility of lower abdominal wall and inguinal ligament reconstruction.

Group 3: Lesions of the pubic and ischiatic branches. These do not require skeletal reconstruction but may benefit from sacroiliac arthrodesis to reduce pain. Abdominal wall and inguinal ligament reconstruction is recommended to prevent visceral herniation. If the resection approaches the medial acetabulum, hip stabilization with autologous iliac crest grafting may be needed.

### Study Limitations

This study has several limitations. First, its retrospective design may introduce selection bias. Second, the sample size is relatively small, limiting the statistical power of subgroup analyses. Third, quantitative measurements of surgical margins were not systematically recorded, preventing detailed analysis of margin thickness. Additionally, follow-up duration varied among patients, and no standardized data on rehabilitation protocols were available. Lastly, as a single-center study, the findings may not be fully generalizable to other clinical settings.

Despite the limitations of this study, histological grade remains a key prognostic factor for both functional outcomes and long-term disease-specific survival in pelvic CSs. OS is significantly influenced by age and tumor grade, while LRFS is more strongly associated with tumor grade. Complication rates appear to correlate more closely with surgical duration.

CSs, however, pose challenges in terms of classification, especially when of low grade. When localized in the pelvis, treatment also becomes technically demanding and is burdened by significant postoperative mortality and less-than-optimal functional outcomes, particularly in cases where the tumor is situated in Enneking and Dunham areas 2, 1, and 4.

A thorough understanding of reconstructive strategies is therefore essential to optimize both oncological safety and postoperative quality of life in this demanding clinical setting.

## Data Availability

Data are available on request in the hospital archives.
